# Level of Knowledge and Risk Factors for Visceral Leishmaniasis in a Mining Area of Minas Gerais State, Brazil

**DOI:** 10.1155/2020/6301310

**Published:** 2020-11-20

**Authors:** Carina Margonari, Júlia Alves Menezes, Gustavo Mayr de Lima Carvalho, Júlia Bahia Miranda, Fabrizio Furtado de Sousa, Felipe Dutra Rêgo, Aldenise Martins Campos, Carolina Cunha Monteiro, Ana Paula Madureira, José Dilermando Andrade Filho

**Affiliations:** ^1^Leishmaniasis Study Group, René Rachou Institute-Oswaldo Cruz Foundation, Belo Horizonte 30190–002, Brazil; ^2^Transdisciplinary Study Group on Health and Environment, René Rachou Institute-Oswaldo Cruz Foundation, Belo Horizonte 30190–002, Brazil; ^3^Divinópolis State University, Divinópolis 35501-170, Brazil; ^4^Department of Biosystems Engineering, Federal University of São João del-Rei, São João del-Rei 36307-352, Brazil

## Abstract

Aiming to optimize and adjust leishmaniasis prevention and control measures for the resident population of Pains, state of Minas Gerais, Brazil, a structured questionnaire containing conceptual questions and questions about household characteristics was used to evaluate knowledge level and exposure risk. A total of 396 individuals were interviewed revealing unscientific and fragmented knowledge about the subject for most of the studied population. The female population was found to have 1.68 times more chance of knowing about the disease than the male population, while highly educated individuals were found to have 2.92 times more chances of knowing about leishmaniasis compared to basic educated individuals. All of the respondents reported the presence of, at least, one risk factor, while ages ≥40 years were considered a protective factor compared to younger ages, indicating that older individuals are more likely to recognize risks and protect themselves against leishmaniasis. These results will contribute to the production of didactic materials for the population with respect to their previous knowledge and will provide a basis for control and prophylactic measures.

## 1. Introduction

Leishmaniasis is considered a globally neglected disease with high morbidity and mortality, with the Americas being one of the main centers of both visceral (VL) and tegumentary forms (ATL). A total of 55,530 human VL cases were reported in the Americas during 2001–2016, representing an annual average of 3,457 cases, with 96% occurring in Brazil [1]. The number of deaths caused by VL by in Brazil has increased since 2012, reaching a lethality rate of 7.9% in 2016—the highest rate compared to other countries of the Americas^1^. The disease manifestations mainly affect both marginalized and impoverished populations and, thus, present a challenge for control programs since great technical-operational and political efforts are required to systematically maintain surveillance actions. The first human case of VL in Brazil was identified in Bahia in 1934 during studies to diagnose yellow fever. In the subsequent years, the role of the domestic dog as a reservoir of the disease was established, as was *Lutzomyia longipalpis* as the vector [2]. At that time, the disease was considered endemic only to the Northeast Region of the country, where it was restricted to rural and wild environments. Since the 1980s, VL has spread throughout Brazil, affecting medium and large municipalities in the northeast, center-west, and southeast regions of the country. The Visceral Leishmaniasis Control Program (PCVL), established in 1963, was based on this context observed in the 19th century and aimed to fight the disease in Brazil. It focused on early diagnosis and treatment of human cases, phlebotomine population control, elimination and/or treatment of infected dogs, and environmental management. Nonetheless, VL expanded into other previously unreached regions, such as the state of Rio Grande do Sul, with its first autochthonous case being recorded in 2009; the state of Paraná, with its first human case in 2015; and the state of Santa Catarina, with the first human and canine cases in 2017 [3, 4].

The shift pattern observed since the 1980s can be attributed to socioenvironmental changes, such as migration from urban centers, urban swelling, precarious living and health conditions, and other modifications to natural environments: deforestation, agricultural expansion, and mining practices [5–9]. Deforestation related to mineral exploration influences the behavior of infectious diseases [10], with land usages such as small-scale mining and agricultural enterprises being identified as factors contributing to higher incidence of diseases such as leishmaniasis [11–14]. On the one hand, the movement of wild mammalian hosts towards human dwellings, as well as the adaptation of vectors to peridomiciliary habitats, is favored by environmental alterations. On the other hand, the domestic dog gained a preponderant epidemiological role in the urban context. The increasingly intimate relationship between dogs and humans permeates controversies and conflicts in the face of canine leishmaniasis cases, which often precede human cases [15].

The current scenario demonstrates that VL control has been flawed since it lacks a popular participation to ensure effective control measures [16, 17]. Although considered in the most recent PCVL guideline, health education has not been used as a capable tool to concretize individual and collective population learning [18]. Several studies have revealed a lack of knowledge by health and education professionals and the general population about leishmaniasis [19–21]. Precarious information about the disease was even presented in didactic books distributed in public school systems [22], collaborating to misinformation of elementary/middle school teachers [23]. This associated with a lack of knowledge among basic care professionals and the general population results in inefficient prevention or control measures [24–28]. Saha et al. [9] argue that ignorance and inadequate education/training in places of greater socioenvironmental vulnerability build “problem areas” where an increase in vector-borne diseases can be seen. In fact, little attention has been given to factors such as patterns of individual mobility, perceptions of disease risk, cultural aspects, and life quality, which may influence leishmaniasis ecoepidemiology [29]. Therefore, studies addressing these factors are necessary in order to determine the reasons for the failure of prevention and control programs.

Thus, the objective of this study was to conduct a survey of VL knowledge level and risk factor awareness in the population of the municipality of Pains, state of Minas Gerais, Brazil. The results are expected to contribute to the understanding of leishmaniasis in the municipality, wherein previous studies documented numerous phlebotomine sand flies associated with a karst environment and high *Leishmania* DNA rates in insects (unpublished data).

## 2. Materials and Methods

### 2.1. Study Guideline

A cross-sectional population-based study was conducted using a structured questionnaire to evaluate the knowledge of leishmaniasis by the population of the municipality of Pains, state of Minas Gerais, Brazil.

**Figure 1:**
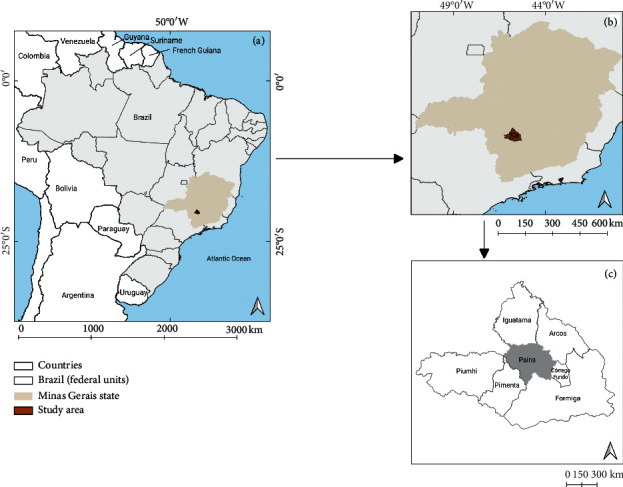
Location of the study area at different regional scales. (a) Overview of the location of the study area on the Brazilian borders, in the state of Minas Gerais, Southeast region. (b) Map showing the location of the study area in which the municipality of Pains is located (Midwest region of the state of Minas Gerais). (c) Municipalities bordering the city of Pains, Minas Gerais, Brazil.

Pains is located in midwestern Minas Gerais, 230 km from Belo Horizonte, the state capital (Figure 1). The municipality is located in a karstic region that also includes other 7 municipalities that are the major limestone producers in Brazil [30, 31]. Pains has high speleological potential, with a great number of subterranean cavities (approximately 1,700) and high paleontological richness [30, 32, 33]. According to the Brazilian Institute of Geography and Statistics (IBGE), the municipality had an estimated 8,270 inhabitants in 2018, an average monthly salary of twice the minimum wage, and a per capita gross domestic product of R$ 34,171.51.

The questionnaire used was based on previous studies [25, 27], with modifications. The instrument contained objective multiple-choice questions, which could have more than one correct answer, divided in Block 1 (knowledge) and Block 2 (peridomiciliary risk factors). Block 3 was related to sociodemographic characteristics of the population. Trained personnel applied the questionnaire in randomly chosen regions of the city. An entomological specimen box was provided to assist with identifying the *Leishmania* vector. It displayed specimens of *Anopheles* sp., *Lutzomyia* sp., *Culex* sp., *Triatoma infestans*, *Musca* sp., and *Aedes* sp. and was presented to respondents to assess whether they were able to identify the Leishmaniasis vector. Participation was voluntary and anonymous.

A proportional stratified-type probabilistic sampling was used according to gender (female and male) and age (15–19 years, 20–24 years, 25–34 years, 35–44 years, 45 years or more). Information used to estimate the size of each stratum was obtained from the 2010 demographic census do IBGE (Instituto Brasileiro de Geografia e Estatística). The representative sample size was 396 people, considering potential losses, and a 10% increase was added to the sample size. The inclusion criterion was to be aged 15 or over. The minimum sample was calculated so that the maximum error of estimate was ±4.98% (or 0.0498) with 95% confidence.

The survey questionnaires were applied by the collaborators/authors of this article at strategic points where there was a large circulation of people, such as door of bank branches, churches, shops, restaurants, and public places (e.g., squares, hospitals, and health centers), always considering the sex/age criterion. After the initial approach, a brief explanation was given about the project under development and the institutions involved in it. To begin the survey, a Free and Informed Consent Form was read and signed. The survey was carried out individually, always with the reading and rereading (if necessary) of the multiple-choice questions/answers by an applicator, who selected the indicated answers to ensure a correct fill out of the questionnaire.

### 2.2. Statistical Analysis

A descriptive analysis was used to create dichotomous indicators to evaluate leishmaniasis knowledge and associated risk factors. Odds ratios, with respective 95% confidence intervals, were calculated to compare respondents regarding their sociodemographic characteristics and their knowledge/risk factor indicators. The model was adjusted for all independent variables. Data were analyzed in Statistical Analysis Software (SAS) version 9.4 for Windows.

### 2.3. Knowledge about Leishmaniasis

The methodology used to evaluate the knowledge of the population is fully described elsewhere [27]. A dichotomous indicator “knowledge about leishmaniasis” was used to estimate the understanding of the multidimensional aspects of the disease, comprising 11 questions, from which respondents were classified into two groups—know or does not know. Only those who answered correctly to all questions were classified as having knowledge about leishmaniasis; the description of the criteria for the correct and incorrect answers is in [27].

### 2.4. Peridomiciliary Risks Factors

A dichotomous indicator “peridomiciliary risk factor” assessed the risk based on the median number of factors reported (median = 9). Households with nine or fewer factors were classified as having lower risk of disease transmission, while households with more than nine factors were classified as having a higher risk. The following answers were considered: positive human case at home; cohousing with domestic animals (dog, cat, or mouse); positive canine case; presence of hematophagous insects at home; *Lutzomyia* sp. vector recognition at home; rodent occurrence at home; existence of a vacant lot near the residence; existence of ecological reserve or dense forest near the residence (500 to 1000 meters); existence of a backyard with plantation; lack of regular cleaning of the peridomicile; animal husbandry (pigsty, chicken coop, kennel, dovecote, and stable) in the vicinity of the residence; watercourse present near home (500 to 1000 meters); and lack of regular garbage collection.

### 2.5. Variables of the Independent Model

Independent variables corresponded to the socioeconomic and demographic characteristics of the population. The covariates used were sex, age (15 to 39 years and ≥40 years), schooling (none to primary, high school, and higher education), family income in minimum wages (<1; between 1 and 2.99; and between 3 and 4.99; >5), and number of residents per household (≤4 or ≥5).

### 2.6. Ethical Issues

This study was submitted and approved by the René Rachou Institute Ethics Committee, Oswaldo Cruz Foundation (IRR/FIOCRUZ), no. 1.351.381.

## 3. Results

A total of 396 individuals living in the municipality of Pains were interviewed. The population profile indicated a prevalence of complete secondary education (68.1%), income between one and three minimum wages (25%), and up to four residents per household (81.22%).

Table 1 shows the percentage of correct answers regarding leishmaniasis knowledge, which included questions about the epidemiological cycle and prevention. None of the respondents answered all 11 questions correctly; only 2.53% answered 10 questions correctly, and 65.9% answered up to six questions correctly. Although 83.6% of the respondents answered to have heard about the disease, a few knew important aspects such as transmission (3.6%), and only 11.4% were able to recognize the vector from the entomological box. Almost half of the interviewees knew the human symptoms (43.2%), while a majority knew how to treat the disease (80.6%). However, 35.6% of respondents never heard about canine visceral leishmaniasis (CVL), and 44.4% were unaware of the procedures recommended by the Ministry of Health (MS) in relation to a case of CVL (euthanasia or treatment with Milteforan®). About 52.4% of respondents said keeping the external environment of the house clean as an effective preventive measure. Approximately 84% indicated the installation of mosquito nets, doors, and window screens; use of repellents; and use of long-sleeved shirts, long pants, socks, and shoes when entering forested areas as individual preventive measures. However, 174 (44%) of the respondents did not know how to control the disease.

Associations between satisfactory knowledge about leishmaniasis and the population socioeconomic characteristics (OR 95% CI) are shown in Table 2. The variables gender and educational level were significant: women had 1.61 times greater chance of knowing about the disease compared to men, while individuals with higher education (high school) were 2.92 times more likely to know about leishmaniasis than those without any education or only basic education.

Regarding peridomiciliary risk factors, about 63% of the respondents reported observing blood-sucking mosquitoes at home, although a few could effectively recognize phlebotomine sand flies (1.3%). The existence of a vacant lot (53.3%), proximity to a green area (51.8%), and yards with plantations and/or fruit trees (47.5%) were the main risk factors reported. Regarding domestic animal ownership, dogs were the most common (37.9%), with 11% of those confirming a positive diagnosis for leishmaniasis. The dichotomous indicator “peridomiciliary risk factors” showed 249 individuals (63.6%) reporting up to nine factors, considered lower risk, and 147 (36.4%) reporting more than nine factors, considered higher risk. All respondents reported the presence of, at least, one risk factor close to the residence. The gross and adjusted odds ratios (95% CI) of the associations between risk factors and population characteristics are showed in Table 2. Age emerged as a protection factor: individuals aged 40 or older presented a 55% lower chance of living with many risk factors at home. The other variables were not significant.

## 4. Discussion

Midwestern Minas Gerais, where the municipality of Pains is located, has experienced an increase in leishmaniasis cases in the recent years. A nearby municipality, Divinópolis, reported a CVL prevalence of 4.6% for an urban area [34] and the occurrence of naturally infected vectors of ATL and VL [35, 36]; about 24 human cases of VL have been reported in the municipality since 2007. A CVL prevalence of 5.8% was observed in the municipality of Formiga, also in the midwestern region, associated with the occurrence of the main vector *Lu. longipalpis*; the first human cases in Formiga were recorded in 2014 [37]. Autochthonous cases of CVL were recently identified in the municipalities of Cláudio and Iguatama [38, 39]. Although unpublished, epidemiological studies developed in Pains also corroborate the dispersal capacity of the disease in the region, since *Leishmania* DNA was found in several sandfly vectors naturally infected by agents of VL or ATL and a CVL prevalence of 9% were found. About 35 human cases of VL and ATL have also been reported in Pains since 1999.

Although epidemiological surveillance acts on the main transmission links—entomological investigation, investigation of human deaths and cases, and surveillance and monitoring of canine cases—the fact is that preventive measures by themselves, as advocated by MS, have not been effective at combating VL in Brazil [40]. On the one hand, knowledge regarding several elements of the transmission chain is still insufficient, as observed in this study. On the other hand, health education actions have often been neglected, although studies point to the importance of popular participation in preventing leishmaniasis [19–21].

A starting point for fostering social mobilization is to understand the knowledge and knowledge gaps regarding the disease and, thus, promote the assembly of knowledge about preventive measures and the epidemiological cycle in the local context. None of the interviewees in Pains correctly answered all the questions about basic disease knowledge, and although 83.6% reported having heard about leishmaniasis, this number is quite lower concerning, CVL- only 35.6%. Barely 3.6% of respondents knew how the disease was transmitted, and 11.4% recognized the vector (*Lu. longipalpis*) presented in the entomological sample box. The same finding was reported in Belo Horizonte, where only between 1 and 3% of the population knew the vectors of leishmaniasis [21]. When researching knowledge among cats and dogs tutors attending a veterinary hospital in São Paulo, Oliveira-Neto et al. [28] observed that 90% knew about the disease, but 34% did not know how to respond to transmission of the disease, and 20% claimed it was transmitted by dog bites. Fragmented knowledge has also been observed by other authors [25, 27], which hinders global understanding of epidemiological links of the disease and reduces the capacity for prevention by the population.

Nearly 52% of the respondents in Pains, Minas Gerais, reported taking precautions regarding possible vector breeding sites—keeping backyards free of leaves and organic matter favorable for sand fly reproduction. Popular participation in keeping places clean, at this point, becomes urgent to sustain the effectiveness of the control measures since these insects are controlled by insecticides sprayed within homes and in peridomiciliary areas, according to MS technical standards [40]. Moreover, municipalities with sporadic transmission as Pains have great difficulty in carrying out the environmental management of the vectors, since the urban perimeter might present large extension and abundant vegetation. In summary, it is counterproductive to unilaterally place blame for ineffective control of any epidemic when partnership between the local population and public policy is the ideal, but distant, scenario for effective combat of the disease [29].

This scenario also applies to issues regarding dogs, which are considered the main reservoir of the disease in urban centers. One of the main recommendations of PCVL regarding canine cases is euthanasia [41]. Currently, in agreement with MS, it is also possible to treat dogs using a protocol and an appropriate drug, Milteforan [42, 43]. However, evaluation of Milteforan treatment for dogs naturally infected with *Leishmania infantum* found that despite improvement of the symptoms, dogs remained parasitologically positive and, thus, remained as reservoirs, contributing to the maintenance of the disease in the environment. Therefore, Milteforan treatment is not recommended for dogs in endemic areas [44]. Euthanasia, despite being widely questioned [45–47], remains a solution for seropositive dogs and is widely debated for several reasons: arguable effectiveness in controlling CVL [47]; nonacceptance of the dog's owner [48, 49]; likely existence of other urban reservoirs such as cats, marsupials, and rodents [50, 51]; and controversy surrounding available diagnostic tests for CVL [52].

These facts, combined with the lack of knowledge in the population about procedures to be taken when faced with a positive dog, make the reservoir and, consequently, the disease difficult to control. The descriptive analysis of the responses of Pains residents revealed that 44% of them were unaware of the recommendations of MS for CVL (i.e., euthanasia or Milteforan treatment). This percentage is significant when considering that, among those who reported having dogs as domestic animals (37.9%), 11% claimed to have received a positive leishmaniasis diagnosis. These findings converge with the results of an investigation of CVL incidence in the municipality, which found 9% of the 114 dogs analyzed to be seropositive (unpublished data). Considering the MS recommendation that establishes disease surveillance and control criteria for municipalities that have CVL incidence above 2%, as well as the social and affective barrier presented by the dog's owner regarding sacrifice, it is evident that a program needs to be initiated that raises awareness of the risks of the disease in accordance with canine reservoir control strategies.

Health surveillance activity that disseminates needful information to the Pains population has been insipient and established in just few municipal health units. Few posters and/or didactic materials are observed in these places. In addition, the role of health professionals (doctors, nurses, health and zoonotic agents, and veterinarians) in disease prevention and information dissemination needs to be strengthened, as knowledge is often exchanged among these workers and the population, whether during home visits or at basic health units. This requires recycling courses to ensure that correct information is spread throughout the population. Studies have revealed gaps in the knowledge of these professionals with regard to leishmaniasis, particularly for topics such as prevention and control [24, 53]. Public media, such as television, radio, and local social networks, have also been underused [54], further restricting the dissemination of information to the population.

From the prevention and risk standpoint, popular misinformation can contribute to the emergence of places favorable for the spread of leishmaniasis. All the respondents reported living with, at least, one risk factor; about 63.6% reported the presence of up to nine risk factors, and 36.4% reported more than nine. The most commonly described risk factors were (i) the presence of hematophagous mosquitoes (63%), although it cannot be determined that these were necessarily sand flies and (ii) the presence of a vacant lot near the residence (53.5%), which is considered a favorable place for vector reproduction and occurrence of synanthropic reservoirs. Other reported risk factors were proximity of green areas (51.8%) and plantation and/or fruit tree in the backyard (47.5%). Many of these conditions are considered favorable for the maintenance of the disease cycle in urban environments, which, when combined with environmental modification resulting from anthropic actions (agriculture, mining, road construction), may directly increase the risk of exposure to infected vectors and reservoirs.

Mining stands out in the context of the municipality of Pains as it is the main economic activity. In addition to having a high environmental impact in the region, mining has already been associated with increases of certain infectious diseases around the world. A study carried out in the Brazilian state of Pará showed an increase in leishmaniasis after the installation of mining operations, with 29% of new human cases being diagnosed in employees or service providers related to the sector [55]. It is likely that the imbalance caused by the destruction of native forest interfered with the balance of the wild cycle of the disease [56]. In the Amazon Forest and French Guiana, mining activities were found to increase the VL risk of workers of these companies [6]. Thus, it is clearly important to consider the direct and indirect impacts of mining on local fauna and flora and its possible effects on the incidence of zoonotic and vector-borne diseases, such as leishmaniasis [57].

Regarding the greater chance of women knowing about the disease in Pains, Minas Gerais, similar results were also observed in the nearby municipality of Formiga [27]. Family care responsibilities is mostly female (i.e., medical appointments, examinations, and hospitalizations), which favors their contact with health professionals and exposure to information related to care and disease prevention [58]. Additionally, the social perception of health services as a feminized space (i.e., frequented and professionalized by women) can make men feel less belonging and hinder their access to healthcare services [59].

The present study found an association between formal education and disease knowledge, as have other studies. Education and income variability acted as obstacles to the acquisition of information on CVL in the municipality of Cotia in the state of São Paulo [60]. A group working in Belo Horizonte, Minas Gerais state capital, found that people who never attended school were eight times more likely to be affected by VL than literate individuals [21]. A systematical review of social inequalities and neglected diseases reported that most studies found a higher chance of VL infection among the lower and less educated socioeconomic strata [61]. Some authors suggest that greater education would increase access to information and contribute to generating practical knowledge, as the educational level influences the quality of life and health promotion [62]. Similarly, disease ignorance was found to be responsible for delaying treatment of confirmed human cases due poor adherence or abandonment [63]. Therefore, the improvement of social and economic conditions could help to reduce inequalities, enhance mitigation of social illness, and stimulate health promotion actions [61].^.^

When it comes to the association between age (over 40 years, in the present study) and lower peridomiciliary risk, the accumulation of life experiences seems to act as a protective factor. The ability to remember facts and procedures to make new associations offers alternatives and solutions, based on accumulated experience built on a daily basis that could be translated into practical knowledge for the prevention of risks associated with leishmaniasis [64]. Knowledge about VL should be recognized for its protective potential since it allows a local population to become conscious and participative in the control of disease and is, thus, an essential factor for VL surveillance and control actions [21].

## 5. Conclusions

The studied population has fragmented knowledge about leishmaniasis and lives in places with numerous risk factors. The lack of concrete, reality-oriented information in the municipality of Pains suggests that the objective of the MS leishmaniasis control and surveillance program for the “development of health education activities with the community” is not being achieved effectively. This leaves no doubt about the necessity for the dissemination of more information on leishmaniasis with the aim of elucidating the complexity of the disease and occurrence of risks in the municipality. Women, greater education, and age were related to knowledge of leishmaniasis and risk factors, suggesting a strategy of awareness and popular participation focused on these most relevant actors for disease control in Pains. To achieve population empowerment, it is necessary to (a) train health teams via a process of continued education; (b) establish associations among different social strata and health, education, and government establishments; and (c) understand the behavior of the disease in Pains. With this, city managers, along with academics, will have the necessary conditions to create and incorporate health education activities focused on leishmaniasis and employ preventive measures considering the prior knowledge, culture, and living and working conditions of the local population.

## Figures and Tables

**Table 1:** The relevant answers from the interviewed residents of the municipality of Pains, Minas Gerais, related to the epidemiological cycle of leishmaniasis.

Knowledge of the population	*n* (%)
Did you hear about leishmaniasis?	
Yes	331 (83.59)
No	65 (16.41)

How is leishmaniasis transmitted?	
Sand fly	129 (32.58)
Incorrect answers	267 (67.42)

Did you recognize the vector?	
* Lutzomyia*	45 (11.36)
Others	351 (88.64)

Who can catch leishmaniasis?	
Correct answers	368 (92.93)
Incorrect answers	28 (7.07)

Do you know what leishmaniasis can cause in humans?	
Fever, weight loss, cough, increased abdominal volume, and skin sores	171 (43.18)
Incorrect answers	225 (56.82)

What do you think needs to be done when a person catches leishmaniasis?	
Immediate treatment	319 (80.56)
Incorrect answers	77 (19.44)

Did you hear about dog leishmaniasis?	
Yes	255 (64.39)
No	141 (35.61)

What do you think needs to be done when a dog catches leishmaniasis?	
Immediate treatment or euthanasia	220 (55.56)
Incorrect answers	176 (44.44)

What can you do to prevent leishmaniasis? (home or work environment)	
Keep the peridomiciliary area clean	208 (52.53)
Incorrect answers	188 (47.47)

What can you do to prevent leishmaniasis? (individual)	
Correct answers	333 (84.09)
Incorrect answers	63 (15.91)

Do you know how leishmaniasis can be controlled?	
Correct answers	222 (56.06)
Incorrect answers	174 (43.94)

**Table 2:** Crude and adjusted odds ratio between knowledge about leishmaniasis/peridomiciliary risks factors and characteristics of the population of the municipality of Pains, Minas Gerais.

Socioeconomic characteristics	Satisfactory knowledge about leishmaniasis		OR (95% CI)		Peridomiciliary risks factors		OR (95% CI)	
	Yes *N* (%)	No *N* (%)	Crude	Adjusted	Higher risk *N* (%)	Lower risk *N* (%)	Crude	Adjusted
*Gender*								
Male	85 (45.45)	102 (54.55)	Ref	Ref	127 (67.91)	60 (32.09)	Ref	Ref
Female	126 (60.29)	83 (39.71)	1.82 (1.22–2.72)	1.61 (1.06–2.45)	125 (59.81)	84 (40.19)	0.70 (0.46–1.06)	0.70 (0.45–1.07)
*Age (years)*								
15–39	104 (53.89)	89 (46.11)	Ref	Ref	140 (72.54)	53 (27.46)	Ref	Ref
≥40	107 (52.71)	96 (47.29)	0.95 (0.64–1.42)	1.37 (0.88–2.13)	112 (55.17)	91 (44.83)	0.47 (0.31–0.71)	0.45 (0.29–0.70)
*Educational level (completed years)*								
None or primary	50 (35.71)	90 (64.29)	Ref	Ref	87 (62.14)	53 (37.86)	Ref	Ref
≥secondary education	161 (62.89)	95 (37.11)	3.05 (1.99–4.68)	2.92 (1.82–4.71)	165 (64.45)	91 (35.55)	1.10 (0.72–1.69)	0.89 (0.55–1.44)
*Income (in minimum wage salaries)*								
≤4.99	96 (46.83)	109 (53.17)	Ref	Ref	126 (61.46)	79 (38.54)	Ref	Ref
≥5.0	115 (60.21)	76 (39.79)	1.72 (1.15–2.56)	1.43 (0.93–2.20)	126 (65.97)	65 (34.03)	1.21 (0.81–1.83)	1.15 (0.74–1.78)
*Number of residents in the household*								
≤4	170 (52.80)	152 (47.20)	Ref	Ref	206 (63.98)	116 (36.02)	Ref	Ref
≥5	41 (55.41)	33 (44.59)	1.11 (0.67–1.85)	1.25 (0.72–2.14)	46 (62.16)	28 (37.84)	0.92 (0.55–1.55)	0.86 (0.50–1.47)

OR: odds ratio; 95% CI: confidence interval of 95% obtained through logistic regression and adjusted by the variables described (396 individuals participated in the analysis).

## Data Availability

The questionnaire data used to support the findings of this study are available from the corresponding author upon request.
